# Tribute to the Memory of Dr. Sprague

**Published:** 1863-02

**Authors:** G. N. Burwell

**Affiliations:** Sec’y pro tem.


### TRIBUTE TO THE MEMORY OF DR. SPRAGUE.

At a special meeting of the Erie County Medical Society to take action upon the death of Dr. A. S. Sprague, the meeting was organized by Dr. Samo, the President, taking the chair, and the appointment of Dr. Burwell as Secretary *pro tem.*

Dr. Samo, on calling the Society to order, remarked at some length upon the talents and great worth of the deceased. Drs. Congar, Loomis, Rochester, Burwell and Wyckoff also severally paid their tribute to his energy and success in the practice of his profession as well as to the great kindness and goodness of his heart.

It was then resolved that a Committee of three be appointed to express the sense of the Society upon its loss in the death of Dr. Sprague. Drs. Loomis, Wyckoff and Burwell were appointed such Committee.

*Resolved*, That we hear with deep feeling, of the death of our associate, Dr. Alden S. Sprague, one of the oldest, most active and influential of the members of this Society. Stricken from among us, many years ago in the midst of his greatest usefulness and fame, and still remembering him as he was in the vigor of his professional career, the Society has sympathized with him in his confinement during his long and grievous illness, and now mourns the final separation of those bonds of friendship and professional brotherhood which were so pleasant in the days of his activity and usefulness. His virtues as a man, his enterprise as a citizen, and his great energy, excellence and talent, as a Physician and Surgeon, placed him at all times, among the first of our citizens, and in the front rank of our profession; while his devotion to the interests of his patients and his ready, self-sacrificing attention to the calls of the indigent and needy, endeared him to the hearts of all, the poor as well as the rich, of those so fortunate as to come under his professional care.

*Resolved*, That we tender our sympathy and condolence to the members of his family, and that a copy of these resolutions be given to them.

*Resolved*, That we will attend his funeral to-morrow at 2 o'clock in the afternoon, in a body.

*Resolved*, That these resolutions be inserted in the Buffalo Medical Journal, and a copy of them be given to the press for publication,

Dr. Wyckoff offered a resolution that the Society appoint the following gentlemen as bearers: Drs. Bristol, Pratt, Barnes, Congar, Samo, Mixer, Lockwood and Burwell.

On motion the Society then adjourned.

G. N. Burwell, *Sec'y pro tem.*

